# Complex mathematical SIR model for spreading of COVID-19 virus with Mittag-Leffler kernel

**DOI:** 10.1186/s13662-021-03470-1

**Published:** 2021-07-03

**Authors:** F. Talay Akyildiz, Fehaid Salem Alshammari

**Affiliations:** Department of Mathematics and Statistics, Faculty of Science, Imam Mohammad ibn Saud Islamic University (IMSIU), Riyadh, Saudi Arabia

**Keywords:** 34A08, 37N30, Coronavirus-19 disease, Complex fractional SIR model, Atangana–Beleanu–Caputo (ABC) derivatives, Fixed-point method, Stability

## Abstract

This paper investigates a new model on coronavirus-19 disease (COVID-19), that is complex fractional SIR epidemic model with a nonstandard nonlinear incidence rate and a recovery, where derivative operator with Mittag-Leffler kernel in the Caputo sense (ABC). The model has two equilibrium points when the basic reproduction number $R_{0} > 1$; a disease-free equilibrium $E_{0}$ and a disease endemic equilibrium $E_{1}$. The disease-free equilibrium stage is locally and globally asymptotically stable when the basic reproduction number $R_{0} <1$, we show that the endemic equilibrium state is locally asymptotically stable if $R_{0} > 1$. We also prove the existence and uniqueness of the solution for the Atangana–Baleanu SIR model by using a fixed-point method. Since the Atangana–Baleanu fractional derivative gives better precise results to the derivative with exponential kernel because of having fractional order, hence, it is a generalized form of the derivative with exponential kernel. The numerical simulations are explored for various values of the fractional order. Finally, the effect of the ABC fractional-order derivative on suspected and infected individuals carefully is examined and compared with the real data.

## Introduction

The coronavirus is a new viral pneumonia, deadly and rapidly spreading infection that has put great panic around the globe since the break out late 2019. Within a short span of time, it affected every country all over the world, which urges every nation take severe action to control the wild spread of the virus as the severity of the disease will harm human life badly.

It is well known that infectious diseases are a massive threat for humans and also for national economies. Proper understanding of a disease dynamics is necessary, which plays a major role for controlling and eventually exterminate the infection in a community. Implementation of the correct applicable strategy against the disease transmission is another challenge. Mathematical modeling of the infectious diseases is one of the key tools in order to handle these challenges. A number of disease models have been established in the past, which enables us to investigate and control the spread of infectious diseases in a better way [1–3].

Regarding COVID-19, from the initial spread of COVID-19, many of the known mathematical models were used, or newly established by many researchers, For example Giordano et al. in [4] proposed the so called SIDARTHE model and concluded that restrictive social-distancing measures will need to be combined with widespread testing and contact tracing to end the ongoing COVID-19 pandemic. At a same time, Alshammari [5] proposed the SEYNHR compartmental model and applied real data for Saudi Arabia and obtained good predictions on a short term. The role of quarantining and isolation to control the spread of COVID-19 is studied by Memon et al. [6], where they used an extended SIR model. They show that quarantine and isolation are effective measures to control the outbreak.

From real data of the infectious disease, we know that the outbreak of the disease within the country or state for time is generally nonlinear, which tells us to design the system where we can study those dynamic nonlinear phenomena. By this system, we can be able to define the transmission of such a contagious disease, which helps us to interpret the remedial measures to stop or contain the spread of the contagious disease.

Most of the present day studies on mathematical modeling of infectious disease are based on integer-order differential equations (IDEs). However, more recently, many authors state that fractional-order models (FDEs) can describe natural phenomena more precisely than the integer-order differential equations because fractional-order derivatives and integrals describe the memory and hereditary properties of different phenomena [7]. Further, the classical IDEs cannot provide the result for non-integer values. In order to overcome the above limitation of integer-order derivatives, different types of fractional-order operators were defined, some of the research can be found in the existing literature [8] and applications of these fractional-order operators are noted in [9], also Sene in [10, 11] consider the Liu et al. [12] chaotic system with Caputo fractional derivative and show the Lyapunov exponent to characterize the nature of chaos and prove the dissipativity of the considered chaotic system.

However, it is known that the classical form of the fractional derivatives with singular kernel may not be suitable to characterize the non-local dynamics in an appropriate manner. In order to overcome this problem, Atangana and Baleanu [13] establish a new model, the so called generalized ABC differential operator involving the Mittag-Leffler (ML) and non-local type kernel, which has an anti-derivative fractional integral operator [14]. This new definition of derivative is shown to be more efficient for the SIR model with generalized incidence rate compared to the other existing fractional models [15] and an exothermic reactions model having a constant heat source in porous media with power problem [16]. More recently, Atangana and Atangana [17] solved the system of ABC fractional partial differential equations to see the side effects of facemasks, which is necessary to protect from COVID-19 and concluded that appropriate facemasks that could help avoid re-inhaling enough CO_2_ should be used every time one is in open air even when alone especially in a windy environment. Later, Khan et al. [18] explored the dynamics of COVID-19 with quarantine and isolations with real statistical cases reported in the mainland China, where the model is developed by using a fractal-fractional derivative in the Atangana–Baleanu sense. They indicate that their results are useful in the early eradication of the disease in the community. Similarly, Sane in [19] also consider the SIR epidemic model with Mittag-Leffler fractional derivative and delay, he uses a Lyapunov direct method and analyzes the global asymptotic stability of the disease-free equilibrium and the endemic equilibrium. Naik et al. [20] proposed an Atangana–Baleanu–Caputo operator for the transmission of COVID-19 epidemic, where they used real data from Pakistan. They show that fractional operators have many advantages over the existing non-integer-order types.

The studies from [13] to [20] provide us with enough motivation to study and analyze a new fractional SIR model involving the ABC operator with complete memory effects. To the best of our knowledge, this is the first time to study a non-local and non-singular derivative operator for the model of the SIR, which has nonlinear incidence and recovery rates, where we also examine the existence and uniqueness results with the Atangana–Baleanu derivative by using a fixed-point method.

The rest of this paper is organized as follows. Some preliminary results are given in Sect. 2. Section 3 is devoted to the new fractional SIR model and its main characteristics. We first derived the solution of the problem and then provided the existence and uniqueness result results in Sect. 4. In the last section, we demonstrate the numerical solutions for Eq. (10) by using the ABC-derivative in a graphical method. We also discussed the necessity of maintaining enough number of hospital beds for controlling of the infectious diseases.

## Preliminary results

We note some background material for the Caputo and Atangana–Baleanu fractional derivatives and Laplace transform are presented [13, 21–23], which we use later.

### Definition 2.1

Mittag-Leffler function is defined as

1 $$\begin{aligned} E_{\alpha } ( x ) = \sum_{k=0}^{\infty } \frac{x^{k}}{\Gamma ( \alpha k+1 )},\quad \alpha >0,\qquad E_{\alpha,\beta } ( x ) = \sum _{k=0}^{\infty } \frac{x^{k}}{\Gamma ( \alpha k+\beta )},\quad \alpha,\beta >0. \end{aligned}$$

### Definition 2.2

Let $f\in C^{n}$ be a function, then the fractional Caputo derivative of order *α* is defined as [13]

2 $$\begin{aligned} {}_{ 0}^{C} D_{t}^{\alpha } \bigl( f ( t ) \bigr) = \int _{0}^{t} \frac{f^{n} ( \tau )}{ ( t-\tau )^{\alpha -n+1}} \,d\tau, \end{aligned}$$

where $\alpha \in ( n-1,n )$ and $n\in\mathbb{ N}$. Obviously, as $\alpha \rightarrow 1$, ${}_{0}^{C} D_{t}^{\alpha } ( f ( t ) ) $ tends to the $f' ( t )$.

### Definition 2.3

Let $f\in H^{1} ( b, b_{1} )$, $\alpha \in ( 0, 1 ] $, then the Atangana–Beleanu–Caputo (ABC) derivative is defined as [13]

3 $$\begin{aligned} {}_{b}^{ ABC} D_{t}^{\alpha } f ( t ) = \frac{B ( \alpha )}{1-\alpha } \int _{b}^{t} f ' ( \tau ) E_{\alpha } \biggl( -\alpha \frac{ ( t-\tau )^{\alpha }}{1-\alpha } \biggr) \,d\tau, \end{aligned}$$

where the term $B ( \alpha )$ is the normalization function with $B ( 0 ) =B ( 1 ) =1$. The associated fractional integral is given by

4 $$\begin{aligned} {}_{0}^{AB} \mathbb{I}_{t}^{\alpha } f ( t ) = \frac{1-\alpha }{B ( \alpha )} f ( t ) + \frac{\alpha }{B ( \alpha ) \Gamma ( \alpha )} \int _{0}^{t} f ( \tau ) ( t-\tau )^{\alpha -1} \,d\tau . \end{aligned}$$

If $\alpha =0$, the initial function is recovered in Eq. (3), we have the ordinary integral for $\alpha =1$.

### Proposition 2.1

*If* $0<\alpha <1$, *we have*

5 $$\begin{aligned} {}_{0}^{AB} \mathbb{I}_{t}^{\alpha } \bigl( {}_{0}^{ABC} D_{t}^{\alpha } f ( t ) \bigr) =f ( t ) -f ( 0 ) E_{\alpha } \bigl( \lambda t^{\alpha } \bigr) - \frac{\alpha }{1-\alpha } f ( 0 ) E_{\alpha,\alpha +1} \bigl( \lambda t^{\alpha } \bigr) =f ( t ) -f ( 0 ) . \end{aligned}$$

### Theorem 2.1

*The Laplace transform of fractional Caputo derivative and Atangana–Beleanu–Caputo* (*ABC*) *derivatives is given by*

6 $$\begin{aligned} \mathcal{L} \bigl\{ {}_{ 0}^{C} D_{t}^{\alpha } \bigl( f ( t ) \bigr) \bigr\} = s^{\alpha } F ( s ) - \sum _{k=0}^{n-1} s^{\alpha -k-1} f^{k} ( 0 ) \end{aligned}$$

*and*

7 $$\begin{aligned} \mathcal{L} \bigl\{ {}_{0}^{ABC} D_{t}^{\alpha } f ( t ) \bigr\} = \frac{B ( \alpha )}{1-\alpha } \frac{s^{\alpha } F ( s ) - s^{\alpha -1} f ( 0 )}{s^{\alpha } + \frac{\alpha }{1-\alpha }}. \end{aligned}$$

### Property 2.1

The inverse Laplace transform of specific functions can be obtained:

8 $$\begin{aligned} \begin{aligned} &(\mathrm{i})\quad \mathcal{L}^{-1} \biggl\{ \frac{s^{\alpha }}{s ( s^{\alpha } +a )} \biggr\} = E_{\alpha } \bigl( -a t^{\alpha } \bigr),\qquad (\mathrm{ii})\quad \mathcal{L}^{-1} \biggl\{ \frac{a}{s ( s^{\alpha } +a )} \biggr\} = 1-E_{\alpha } \bigl( -a t^{\alpha } \bigr),\\ & (\mathrm{iii})\quad \mathcal{L}^{-1} \biggl\{ \frac{1}{ ( s^{\alpha } +a )} \biggr\} = t^{\alpha -1} E_{\alpha } \bigl( -a t^{\alpha } \bigr). \end{aligned} \end{aligned}$$

## Mathematical model and discussion

**Figure 1:**
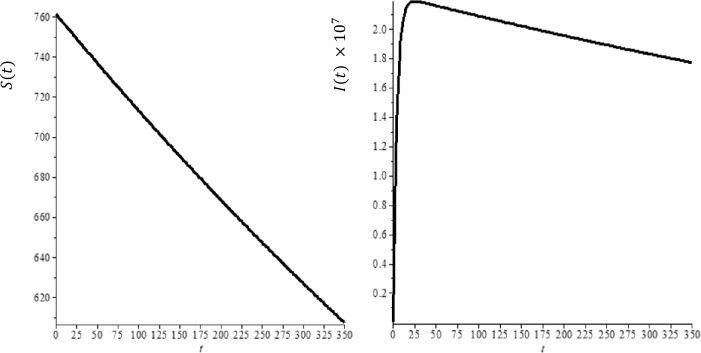
Time development of susceptible (**a**) and infected (**b**) individuals for integer of order derivatives, $b=100$ and $R_{0} =2.618$

Let us consider the total population $N ( t )$ to consist of three sub-categories: susceptible $S ( t )$, infected $I ( t )$ and recovered $R ( t )$ individuals $( SIR )$, where $N ( t ) =S ( t ) +I ( t ) +R ( t )$ as seen in Fig. 1. There are many factors involved in modeling the infectious diseases and these factors substantially affect the dynamical behavior of the models such as incident rate and recovery rate. In the classical SIR model, incident rate $\beta \frac{S ( t ) I ( t )}{N ( t )}$ and linear recovery rate $\mu I ( t )$ are used. However, as for COVID-19, many infectious diseases show multiple peaks or periodic oscillations during the outbreak or in progress. Therefore, several nonlinear incidence rates have been suggested since they can produce rich dynamics for the epidemic models [24, 25]. Liu et al. [26] proposed the following form of nonlinear saturated incidence rate to include the effect of behavioral changes:

9 $$\begin{aligned} G ( I ) S= \frac{\beta ^{*} I^{l}}{1+ k^{*} I^{h}}, \end{aligned}$$

where $\beta ^{*},l,h, k^{*} >0$. This model is due to Ruan et al. [27, 28]. More recently, a more general form was considered by Rao et al. [29].

On the other hand, in view of the diversity of public resources, it is not enough to study the effect of treatment capacity on a large scale at this time, if we want to further explore the influence of hospital bed number on disease transmission. Then we need to define the nonlinear recovery rate introduced by [30]

10 $$\begin{aligned} \Pi ( I,b ) = \biggl( \alpha _{0} + ( \alpha _{1} - \alpha _{0} ) \frac{b}{b+I} \biggr), \end{aligned}$$

where $\alpha _{1}$ is the maximum per capita recovery rate, when health care resources suffice and there are few infectious individuals, $\alpha _{0}$ is the minimum per capita recovery rate due to lack of clinical resources, *b* is the ratio between the number of hospital bed and the population, all the above parameters are non-negative. It has the following properties: (i) $\frac{\partial \Pi ( I,b )}{\partial I} <0, \lim_{I\rightarrow \infty } \Pi ( I,b ) = \alpha _{0}$ and $\lim_{I\rightarrow 0} \Pi ( I,b ) = \alpha _{1}$, i.e., $\Pi ( I,b )$ is a decreasing function of *I*. (ii) $\frac{\partial \Pi ( I,b )}{\partial b} >0, \lim_{b\rightarrow \infty } \Pi ( I,b ) = \alpha _{1}$ and $\lim_{b\rightarrow 0} \Pi ( I,b ) = \alpha _{0}$, i.e., $\Pi ( I,b )$ is an increasing function of *b*.

Here, we assume individuals move from compartment *S* to *I* with transmission rates $\frac{\beta IS}{k+I} ( l=h=1\text{ in Eq. } ( 9 ) )$, and when an individual is infected, then the individual either recovers at a rate $\Pi ( I,b ) = ( \alpha _{0} + ( \alpha _{1} - \alpha _{0} ) \frac{b}{b+I} )$ or dies at the rate of *γI*. Then this complex SIR model can be represented by following nonlinear system of equations:

11 $$\begin{aligned} \left .\textstyle\begin{array}{l} \frac{dS}{dt} =A - \frac{\beta IS}{k+I} -\mu S\\ \frac{dI}{dt} = \frac{\beta IS}{k+I} - ( \alpha _{0} + ( \alpha _{1} - \alpha _{0} ) \frac{b}{b+I} ) I- ( \gamma +\mu ) I\\ \frac{dR}{dt} = ( \alpha _{0} + ( \alpha _{1} - \alpha _{0} ) \frac{b}{b+I} ) I-\mu R \end{array}\displaystyle \right \}, \end{aligned}$$

where $A=n\times N, N$ refer to total number of people and *n* is the birth rate, *μ* is a positive constant representing the natural death of the population, *γ* is a positive constant representing the disease induced death rate, $\alpha _{0}$ is the minimum per capita recovery rate due to the function of basic clinical resources and $\alpha _{1}$ is the maximum per capita recovery rate due to the sufficient health care resource and few infectious individuals as well as the inherent property of a specific disease.

The model in (11) is not capable of representing the influence of memory effects with respect to time derivative. In order to account for the memory effect, in addition, we modify the fractional operator by an auxiliary parameter *σ*, having the dimension of second, to ensure that the right- and left-hand sides of the resultant equation possess the same dimension $s^{-1}$. Consequently, we can reformulate the system in (9) by using the ABC derivatives as

12 $$\begin{aligned} \left .\textstyle\begin{array}{l} \frac{1}{\sigma ^{\alpha -1}} {}_{0}^{ABC} D_{t}^{\alpha } S ( t ) =A- \frac{\beta IS}{k+I} -\mu S\\ \frac{1}{\sigma ^{\alpha -1}} {}_{0}^{ABC} D_{t}^{\alpha } I ( t ) = \frac{\beta IS}{k+I} - ( \alpha _{0} + ( \alpha _{1} - \alpha _{0} ) \frac{b}{b+I} ) I- ( \gamma +\mu ) I\\ \frac{1}{\sigma ^{\alpha -1}} {}_{0}^{ABC} D_{t}^{\alpha } R ( t ) = ( \alpha _{0} + ( \alpha _{1} - \alpha _{0} ) \frac{b}{b+I} ) I-\mu R\\ \text{with } S ( 0 ) = S_{0},I ( 0 ) = I_{0},R ( 0 ) = R_{0} \end{array}\displaystyle \right \}, \end{aligned}$$

where $0<\alpha <1 $. In the first two equations in (12), $R ( t )$ does not appear, it is therefore assumed the first two equations are sufficient to handle the full system. We modify the fractional operator by an auxiliary parameter *σ*, having the dimension of day, to ensure that the right- and left-hand sides of the resultant equation possess the same dimension $s^{-1}$

### Non-negative solution

In this section, we show that the feasibility region of the system (12) is given by the closed set

13 $$\begin{aligned} \Omega = \biggl\{ ( S,I,T ):S+I+R\leq \frac{A}{\mu } \biggr\} , \end{aligned}$$

which is positively invariant.

#### Lemma 3.1

*The closed set defined in* (13) *is positively invariant with respect to the fractional model* (12).

#### Proof

Adding all three equations in (12), we have

14 $$\begin{aligned} {}_{0}^{ABC} D_{t}^{\alpha } N ( t ) = \sigma ^{\alpha -1} ( A-\mu N-\gamma I ) \leq \sigma ^{\alpha -1} ( A-\mu N ). \end{aligned}$$

Applying the Laplace transform on both sides, we obtain

15 $$\begin{aligned} \mathcal{L} \bigl\{ {}_{0}^{ABC} D_{t}^{\alpha } N ( t ) \bigr\} + \hat{\mu } \mathcal{L} \bigl\{ N ( t ) \bigr\} \leq \mathcal{L} \{ \hat{A} \},\quad \hat{\mu } = \sigma ^{\alpha -1} \mu \text{ and } \hat{A} = \sigma ^{\alpha -1} A . \end{aligned}$$

Using Theorem 2.1 and the properties of the Laplace transform, we got

16 $$\begin{aligned} \frac{B ( \alpha )}{1-\alpha } \frac{s^{\alpha } N ( s ) - s^{\alpha -1} N ( 0 )}{s^{\alpha } + \frac{\alpha }{1-\alpha }} + \hat{\mu } N(s)\leq \frac{\hat{A}}{s}. \end{aligned}$$

Hence

17 $$\begin{aligned} N ( s ) \leq \frac{B ( \alpha ) N(0) s^{\alpha -1}}{ ( B ( \alpha ) + \hat{\mu } ( 1-\alpha ) ) s^{\alpha } +\alpha \hat{\mu }} + \frac{\hat{A} ( s^{\alpha } ( 1-\alpha ) +\alpha )}{s ( ( B ( \alpha ) + \hat{\mu } ( 1-\alpha ) ) s^{\alpha } +\alpha \hat{\mu } )} \end{aligned}$$

now, applying the inverse Laplace transform and using Property 2.1, we find

18 $$\begin{aligned} N(t)\leq {}&\frac{B ( \alpha ) N(0)}{ ( B ( \alpha ) + \hat{\mu } ( 1-\alpha ) )} E_{\alpha } \bigl( -m t^{\alpha } \bigr) + \frac{\hat{A} ( 1-\alpha )}{ ( B ( \alpha ) + \hat{\mu } ( 1-\alpha ) )} E_{\alpha } \bigl( -m t^{\alpha } \bigr) \\ &{} + \frac{\hat{A}}{\hat{\mu }} \bigl( 1- E_{\alpha } \bigl( -m t^{\alpha } \bigr) \bigr), \end{aligned}$$

where $m= \frac{\alpha \hat{\mu }}{ ( B ( \alpha ) + \hat{\mu } ( 1-\alpha ) )}$, since the Mittag-Leffler function has an asymptotic behavior, we obtain $N ( t ) \leq \frac{\hat{A}}{\hat{\mu }} = \frac{A}{\mu } $ as $t\rightarrow \infty $. Hence, a solution of the fractional model (12) stays in Ω for every $t>0$. Consequently, the closed set is a positively invariant set regarding the fractional model (12). □

### Equilibrium points, basic reproduction number and local stability of equilibrium points

The equilibrium points of the system are the zeros of second side of Eq. (12), obviously, disease-free equilibrium point i.e., $I=0$ and $S=A/\mu $ or $E_{0} = ( \frac{A}{\mu },0 )$. Therefore, the model (12) has a threshold parameter $R_{0}$, known as the basic reproduction number, this is defined as the number of secondary infections produced by a single infection in a completely susceptible population. It is not difficult to write the reproduction number for the disease-free equilibrium point of Eq. (12) as [5]

19 $$\begin{aligned} R_{0} = \frac{\beta A}{k\mu ( \alpha _{1} +\gamma +\mu )} . \end{aligned}$$

The existence of the endemic equilibrium point(s) can be determined by the relation

20 $$\begin{aligned} S^{*} = \frac{ ( \alpha _{0} I^{*} +b \alpha _{1} +b\gamma +b\mu +\gamma I^{*} ) ( k+ I^{*} )}{\beta ( b+ I^{*} )}, \end{aligned}$$

where $I^{*}$ is the solution of the following equation:

21 $$\begin{aligned} A_{1} I^{*2} + A_{2} I^{*} + A_{3} =0 . \end{aligned}$$

Here, the coefficients are given by

22 $$\begin{aligned} \left . \textstyle\begin{array}{l} A_{1} = \mathrm{b} ( \beta + \frac{\mu }{k} ) ( \alpha _{0} +\gamma +\mu )\\ A_{2} =b ( \beta + \frac{\mu }{k} ) ( \alpha _{1} +\gamma +\mu ) +\mu ( \alpha _{1} +\gamma +\mu ) ( 1- R_{0} ) -\mu ( \alpha _{1} - \alpha _{0} )\\ A_{3} =\mu ( \alpha _{1} +\gamma +\mu ) ( 1- R_{0} ) \end{array}\displaystyle \right \}. \end{aligned}$$

The solution of quadratic equation in (21) is given by

23 $$\begin{aligned} I^{*} = \frac{- A_{2} \mp \sqrt{A_{2}^{2} -4 A_{1} A_{3}}}{2 A_{1}}. \end{aligned}$$

Then we have the three cases of $R_{0}$

∗: If $R_{0} <1$, so, $A_{1}$ and $A_{3} >0$, if $b\geq \frac{\mu ( \alpha _{1} - \alpha _{0} )}{b ( \beta + \frac{\mu }{k} ) ( \alpha _{1} +\gamma +\mu ) ( 1+\mu ) ( 1- R_{0} )}$ no endemic equilibrium, if $b< \frac{\mu ( \alpha _{1} - \alpha _{0} )}{b ( \beta + \frac{\mu }{k} ) ( \alpha _{1} +\gamma +\mu ) ( 1+\mu ) ( 1- R_{0} )}$, we have no endemic equilibrium for $A_{2}^{2} -4 A_{1} A_{3} <0$, one positive endemic equilibrium for $A_{2}^{2} -4 A_{1} A_{3} =0$ and two endemic equilibrium points for $A_{2}^{2} -4 A_{1} A_{3} >0$.
∗: If $R_{0} =1$, then, if $b< \frac{\mu ( \alpha _{1} - \alpha _{0} )}{b ( \beta + \frac{\mu }{k} ) ( \alpha _{1} +\gamma +\mu ) ( 1+\mu ) ( 1- R_{0} )}$, then system unique endemic equilibrium point.
∗: If $R_{0} >1$, then, if $b< \frac{\mu ( \alpha _{1} - \alpha _{0} )}{b ( \beta + \frac{\mu }{k} ) ( \alpha _{1} +\gamma +\mu ) ( 1+\mu ) ( 1- R_{0} )}$, then system unique endemic equilibrium point.

#### Theorem 3.1

*The disease*-*free equilibrium point* $\mathrm{E}_{0}$ *is locally asymptotically stable and if* $\mathrm{R}_{0} <1$ *then it is unstable if* $\mathrm{R}_{0} >1$.

#### Proof

The jacobian matrix corresponding to system (12) is

24 $$\begin{aligned} J ( S,I ) = \begin{pmatrix} - \frac{\beta I}{k+I} -\mu & \frac{k\beta S}{ ( k+I )^{2}}\\ \frac{\beta I}{k+I} & \frac{k\beta S}{ ( k+I )^{2}} + \frac{ ( \alpha _{1} - \alpha _{0} ) b^{2}}{ ( b+I )^{2}} - ( \alpha _{0} +\gamma +\mu ) \end{pmatrix} . \end{aligned}$$

Then the solution of characteristic equation associated to $J ( \frac{A}{\mu },0 )$ is given by

25 $$\begin{aligned} \lambda _{1} =-\mu, \lambda _{2} = \frac{1}{ ( \alpha _{1} +\gamma +\mu )} ( R_{0} -1 ) . \end{aligned}$$

Hence, if $R_{0} <1$, the disease-free equilibrium point $\mathrm{E}_{0}$ is locally asymptotically stable, if $\mathrm{R}_{0} >1$, it is unstable. □

#### Theorem 3.2

*A sufficient condition for the endemic equilibrium* $E_{1} = ( S^{*}, I^{*} )$ *to be locally asymptotically stable is* $N_{1} <0$ *and* $N_{2} >0$, *where*

26 $$\begin{aligned} N_{1} =\operatorname{Tr} \bigl( J \bigl( S^{*}, I^{*} \bigr) \bigr),\qquad N_{2} =\operatorname{Det} \bigl( J \bigl( S^{*}, I^{*} \bigr) \bigr). \end{aligned}$$

#### Proof

See the book of Chou and Friedman [31], we here note that

27 $$\begin{aligned} - \frac{k \beta ^{\alpha } S}{ ( k+I )^{2}} + \frac{ ( ( \beta +\mu ) I+\mu k ) ( \alpha _{1} +\gamma +\mu )}{ ( k+I )} &< \operatorname{Det} \bigl( J \bigl( S^{*}, I^{*} \bigr) \bigr) \\ &< - \frac{k\beta S}{ ( k+I )^{2}} + \frac{ ( ( \beta +\mu ) I+\mu k ) \beta A}{\mu k R_{0}}. \end{aligned}$$

Therefore, local asymptotic stability of the endemic equilibrium point is a function of $R_{0}$ and the number of available hospital beds $( b )$. □

#### Theorem 3.3

*For the system* (12), *if* $R_{0} <1$, *i*.*e*. $\beta A< k\mu ( \alpha _{1} +\gamma +\mu )$, *the disease*-*free equilibrium* $E_{0} = ( \frac{A}{\mu },0 )$ *is globally asymptotically stable*.

#### Proof

Consider the Lyapunov function $V ( t ) =I$ in $R^{2+}$, Calculating the time fractional derivative of the $V ( t )$, we obtain

28 $$\begin{aligned} {}_{0}^{ABC} D_{t}^{\alpha } V ( t ) = {}_{0}^{ABC} D_{t}^{\alpha } I ( t ). \end{aligned}$$

Utilizing the system (12), we have

29 $$\begin{aligned} {}_{0}^{ABC} D_{t}^{\alpha } V ( t )& = \lim _{S\rightarrow \frac{A}{\mu },I\rightarrow 0} \frac{\beta IS}{k+I} - \biggl( \alpha _{0} + ( \alpha _{1} - \alpha _{0} ) \frac{b}{b+I} \biggr) I- ( \gamma +\mu ) I \\ &= \lim_{S\rightarrow \frac{A}{\mu },I\rightarrow 0} \biggl( \frac{\beta S}{k+I} - \biggl( \alpha _{0} + ( \alpha _{1} - \alpha _{0} ) \frac{b}{b+I} \biggr) - ( \gamma +\mu ) \biggr) \lim_{S\rightarrow \frac{A}{\mu },I\rightarrow 0} I \\ &= \bigl( \beta A-k\mu ( \alpha _{1} +\gamma +\mu ) \bigr) \lim _{S\rightarrow \frac{A}{\mu },I\rightarrow 0} I \\ &=k\mu ( \alpha _{1} +\gamma +\mu ) ( R_{0} -1 ) \lim_{S\rightarrow \frac{A}{\mu },I\rightarrow 0} I\leq 0 . \end{aligned}$$

The Lyapunov–Lasalle theorem implies that solutions in Ω approach the largest positively in-variant subset of the set $V ' =0$, i.e., the plane $I =0$. In this plane, $S \rightarrow \frac{A}{d}$ as $t \rightarrow \infty $. Thus all solutions in the plane $I =0$ go to the disease-free equilibrium $E_{0}$. Therefore $E_{0}$ is globally asymptotically stable. □

## Existence and uniqueness of the solution

Applying the *AB*-fractional integral on both sides of (12), we obtain

30 $$\begin{aligned} \left .\textstyle\begin{array}{l} {}_{0}^{AB} \Sigma _{t}^{\alpha } ( {}_{0}^{ABC} D_{t}^{\alpha } S ( t ) ) = {}_{0}^{AB} \Sigma _{t}^{\alpha } \sigma ^{\alpha -1} \lceil A- \frac{\beta IS}{k+I} -\mu S \rceil \\ {}_{0}^{AB} \Sigma _{t}^{\alpha } ( {}_{0}^{ABC} D_{t}^{\alpha } I ( t ) ) = {}_{0}^{AB} \Sigma _{t}^{\alpha } \sigma ^{\alpha -1} \lceil \frac{\beta IS}{k+I} - ( \alpha _{0} + ( \alpha _{1} - \alpha _{0} ) \frac{b}{b+I} ) I- ( \gamma +\mu ) I \rceil \end{array}\displaystyle \right \} . \end{aligned}$$

By using Proposition 2.1, we have

31 $$\begin{aligned} \left .\textstyle\begin{array}{l}S ( t ) - S_{0} = {}_{0}^{AB} \Sigma _{t}^{\alpha } \sigma ^{\alpha -1} \lceil A- \frac{\beta IS}{k+I} -\mu S \rceil \\ I ( t ) - I_{0} = {}_{0}^{AB} \Sigma _{t}^{\alpha } \sigma ^{\alpha -1} [ \frac{\beta IS}{k+I} - ( \alpha _{0} + ( \alpha _{1} - \alpha _{0} ) \frac{b}{b+I} ) I- ( \gamma +\mu ) I ] \end{array}\displaystyle \right \}. \end{aligned}$$

Using Eq. (3) in the above, we find

32 $$\begin{aligned} S ( t ) - S_{0} ={}& \frac{1-\alpha }{X ( \alpha )} M_{1} \bigl( \alpha,t,S ( t ),I ( t ) \bigr) \\ &{} + \frac{\alpha }{X ( \alpha ) \Gamma ( \alpha )} \int _{0}^{t} M_{1} \bigl( \alpha,\tau,S ( \tau ),I ( \tau ) \bigr) ( t - \tau )^{\alpha - 1} \,d\tau \end{aligned}$$

and

33 $$\begin{aligned} I ( t ) - I_{0} ={}& \frac{1-\alpha }{X ( \alpha )} M_{2} \bigl( \alpha,t,S ( t ),I ( t ) \bigr) \\ &{} + \frac{\alpha }{X ( \alpha ) \Gamma ( \alpha )} \int _{0}^{t} M_{2} \bigl( \alpha,\tau,S ( \tau ),I ( \tau ) \bigr) ( t - \tau )^{\alpha - 1} \,d\tau, \end{aligned}$$

where

34 $$\begin{aligned} \begin{aligned} &M_{1} \bigl( \alpha,t,S ( t ),I ( t ) \bigr) = \sigma ^{\alpha -1} \biggl( A- \frac{\beta IS}{k+I} -\mu S \biggr)\quad \text{and}\\ & M_{2} \bigl( \alpha,t,S ( t ),I ( t ) \bigr) = \sigma ^{\alpha -1} \biggl( \frac{\beta IS}{k+I} - \biggl( \alpha _{0} + ( \alpha _{1} - \alpha _{0} ) \frac{b}{b+I} \biggr) I- ( +\mu ) I \biggr). \end{aligned} \end{aligned}$$

The above system of Eqs. (32)–(33) is the solution of the systems of Eq. (12),

35 $$\begin{aligned} \left . \textstyle\begin{array}{l} T ( S ( t ) ) = \frac{1-\alpha }{X ( \alpha )} M_{1} ( \alpha,t,S ( t ),I ( t ) ) + \frac{\alpha }{X ( \alpha ) \Gamma ( \alpha )} \int _{0}^{t} M_{1} ( \alpha,\tau,S ( \tau ),I ( \tau ) ) ( t - \tau )^{\alpha - 1} \,d\tau \\ T ( I ( t ) ) = \frac{1-\alpha }{X ( \alpha )} M_{2} ( \alpha,t,S ( t ),I ( t ) ) + \frac{\alpha }{X ( \alpha ) \Gamma ( \alpha )} \int _{0}^{t} M_{2} ( \alpha,\tau,S ( \tau ),I ( \tau ) ) ( t - \tau )^{\alpha - 1} \,d\tau \end{array}\displaystyle \right \} . \end{aligned}$$

Let $C [ 0, T ]$ with subnorm be a Banach space of the real valued continuous functions. $M = C [ 0, T ] \times C [ 0, T ]$ with the norm $\Vert ( S, I ) \Vert = \Vert S \Vert + \Vert I \Vert , \Vert S \Vert = \sup_{t\in [ 0, T ]} \vert S ( t ) \vert $ and $\Vert I \Vert = \sup_{t\in [ 0, T ]} \vert I ( t ) \vert $.

### Theorem 4.1

*The kernels*, $M_{1} ( \alpha,t,S ( t ),I ( t ) )$ *and* $M_{2} ( \alpha,t,S ( t ),I ( t ) )$, *satisfy the Lipschitz condition and contraction if the following inequality holds*:

36 $$\begin{aligned} 0\leq L_{1} < 1 \quad\textit{and}\quad 0\leq L_{2} < 1. \end{aligned}$$

### Proof

Consider $M_{1} ( \alpha,t,S ( t ),I ( t ) ) = A^{\alpha } - \frac{\beta ^{\alpha } IS}{k+I} - \mu ^{\alpha } S$, Let $S ( t )$ and $S^{*} ( t )$ be two functions, then we have

37 $$\begin{aligned} \bigl\Vert M_{1} ( \alpha,t,S,I ) - M_{1} \bigl( \alpha,t, S^{*},I \bigr) \bigr\Vert &= \sigma ^{\alpha -1} \biggl\Vert - \frac{\beta IS}{k+I} -\mu S+ \frac{\beta I S^{*}}{k+I} +\mu S^{*} \biggr\Vert \\ &\leq L_{1} \bigl\Vert S- S^{*} \bigr\Vert , \end{aligned}$$

where $L_{1} = \Vert \mu ^{\alpha } + \frac{\beta ^{\alpha } I}{k+I} \Vert $ is bounded since $I(t)$ is bounded, hence the Lipschitz condition is satisfied. If $0\leq L_{1} <1$, then $M_{1} ( \alpha,t,S ( t ),I ( t ) )$ becomes a contraction mapping. Similarly, for $M_{2} ( \alpha,t,S ( t ),I ( t ) )$, we find

38 $$\begin{aligned} \bigl\Vert M_{2} ( \alpha,t,S,I ) - M_{2} \bigl( \alpha,t,S, I^{*} \bigr) \bigr\Vert \leq L_{2} \bigl\Vert I- I^{*} \bigr\Vert , \end{aligned}$$

where

39 $$\begin{aligned} L_{2} = \sigma ^{\alpha -1} \biggl\Vert \frac{k\beta S}{ ( k+I ) ( k+ I^{*} )} - \frac{b^{2} ( \alpha _{1} - \alpha _{0} )}{ ( b+I ) ( b+ I^{*} )} - \alpha _{0} \biggr\Vert . \end{aligned}$$

Since $S ( t ), I ( t )$ and $I^{*} ( t )$ are bounded, hence the Lipschitz condition is satisfied, if $0\leq L_{2} <1$, $M_{2} ( \alpha,t,S ( t ),I ( t ) )$ becomes a contraction mapping. □

### Theorem 4.2

*Suppose that the following condition holds*:

40 $$\begin{aligned} \frac{1-\alpha }{X ( \alpha )} L_{i} + \frac{1}{X ( \alpha ) \Gamma ( \alpha )} L_{i} T^{\alpha } < 1,\quad i=1,2. \end{aligned}$$

*Then the fractional epidemic model* (12) *has a unique solution for* $t\in [ 0,T ]$.

### Proof

Recursively, the expressions in (35) can be written as

41 $$\begin{aligned} S_{n} ( t ) ={}& \frac{1-\alpha }{X ( \alpha )} M_{1} \bigl( \alpha,t, S_{n-1} ( t ), I_{n-1} ( t ) \bigr) \\ &{}+ \frac{\alpha }{X ( \alpha ) \Gamma ( \alpha )} \int _{0}^{t} M_{1} \bigl( \alpha,\tau, S_{n-1} ( \tau ), I_{n-1} ( \tau ) \bigr) ( t - \tau )^{\alpha - 1} \,d\tau , \end{aligned}$$

42 $$\begin{aligned} I_{n} ( t ) = {}&\frac{1-\alpha }{X ( \alpha )} M_{2} \bigl( \alpha,t, S_{n-1} ( t ), I_{n-1} ( t ) \bigr) \\ &{} + \frac{\alpha }{X ( \alpha ) \Gamma ( \alpha )} \int _{0}^{t} M_{2} \bigl( \alpha,\tau, S_{n-1} ( \tau ), I_{n-1} ( \tau ) \bigr) ( t - \tau )^{\alpha - 1} \,d\tau . \end{aligned}$$

As usual taking the difference of successive approximations, we obtain

43 $$\begin{aligned} \Pi _{n}^{S} ={}&S_{n} ( t ) - S_{n-1} ( t ) \\ = {}&\frac{1-\alpha }{X ( \alpha )} \bigl[ M_{1} \bigl( \alpha,t, S_{n-1} ( t ), I_{n-1} ( t ) \bigr) - M_{1} \bigl( \alpha,t, S_{n-1} ( t ), I_{n-1} ( t ) \bigr) \bigr] \\ &{}+ \frac{\alpha }{X ( \alpha ) \Gamma ( \alpha )} \int _{0}^{t} \bigl[ M_{1} \bigl( \alpha, \tau, S_{n-1} ( \tau ), I_{n-1} ( \tau ) \bigr) \\ &{}- M_{1} \bigl( \alpha,\tau, S_{n-1} ( \tau ), I_{n-1} ( \tau ) \bigr) \bigr] ( t - \tau )^{\alpha - 1} \,d\tau , \end{aligned}$$

44 $$\begin{aligned} \Pi _{n}^{I} ={}&I_{n} ( t ) - I_{n-1} ( t ) \\ = {}&\frac{1-\alpha }{X ( \alpha )} \bigl[ M_{2} \bigl( \alpha,t, S_{n-1} ( t ), I_{n-1} ( t ) \bigr) - M_{2} \bigl( \alpha,t, S_{n-1} ( t ), I_{n-1} ( t ) \bigr) \bigr] \\ &{}+ \frac{\alpha }{X ( \alpha ) \Gamma ( \alpha )} \int _{0}^{t} \bigl[ M_{2} \bigl( \alpha, \tau, S_{n-1} ( \tau ), I_{n-1} ( \tau ) \bigr) \\ &{}- M_{2} \bigl( \alpha,\tau, S_{n-1} ( \tau ), I_{n-1} ( \tau ) \bigr) \bigr] ( t - \tau )^{\alpha - 1} \,d\tau. \end{aligned}$$

It is not difficult to see that $S_{n} ( t ) = \sum_{i}^{n} \Pi _{i}^{S}$ and $I_{n} ( t ) = \sum_{i}^{n} \Pi _{i}^{I}$, taking the sup norm on both sides, and using the Lipschitz condition on kernel functions and triangle inequality, we obtain

45 $$\begin{aligned} \bigl\Vert \Pi _{n}^{S} \bigr\Vert \leq{}& \frac{1-\alpha }{X ( \alpha )} L_{1} \bigl\Vert \Pi _{n-1}^{S} \bigr\Vert + \frac{\alpha L_{1}}{X ( \alpha ) \Gamma ( \alpha )} \int _{0}^{t} \bigl\Vert \Pi _{n-1}^{S} \bigr\Vert ( t - \tau )^{\alpha - 1} \,d\tau \\ ={}& \frac{1-\alpha }{X ( \alpha )} L_{1} \bigl\Vert \Pi _{n-1}^{S} \bigr\Vert + \frac{t^{\alpha }}{X ( \alpha ) \Gamma ( \alpha )} L_{1} \bigl\Vert \Pi _{n-1}^{S} \bigr\Vert , \end{aligned}$$

46 $$\begin{aligned} \bigl\Vert \Pi _{n}^{I} \bigr\Vert \leq{}& \frac{1-\alpha }{X ( \alpha )} L_{2} \bigl\Vert \Pi _{n-1}^{I} \bigr\Vert + \frac{\alpha L_{2}}{X ( \alpha ) \Gamma ( \alpha )} \int _{0}^{t} \bigl\Vert \Pi _{n-1}^{I} \bigr\Vert ( t - \tau )^{\alpha - 1} \,d\tau \\ ={}& \frac{1-\alpha }{X ( \alpha )} L_{2} \bigl\Vert \Pi _{n-1}^{I} \bigr\Vert + \frac{t^{\alpha }}{X ( \alpha ) \Gamma ( \alpha )} L_{2} \bigl\Vert \Pi _{n-1}^{I} \bigr\Vert . \end{aligned}$$

Starting from $n=1$, we obtain by back substitution from (30) and (31), respectively,

47 $$\begin{aligned} \bigl\Vert \Pi _{n}^{S} \bigr\Vert \leq \bigl\Vert \Pi _{1}^{S} \bigr\Vert \biggl( \frac{1-\alpha }{X ( \alpha )} L_{1} + \frac{1}{X ( \alpha ) \Gamma ( \alpha )} L_{1} T^{\alpha } \biggr)^{n - 1} \end{aligned}$$

and

48 $$\begin{aligned} \bigl\Vert \Pi _{n}^{I} \bigr\Vert \leq \bigl\Vert \Pi _{1}^{I} \bigr\Vert \biggl( \frac{1-\alpha }{X ( \alpha )} L_{2} + \frac{1}{X ( \alpha ) \Gamma ( \alpha )} L_{2} T^{\alpha } \biggr)^{n - 1} . \end{aligned}$$

It is clear from our hypothesis that $\Vert \Pi _{n}^{S} \Vert \rightarrow 0$ and $\Vert \Pi _{n}^{I} \Vert \rightarrow 0$ as $n\rightarrow \infty $. We obtain a convergent series as follows:

49 $$\begin{aligned} \bigl\Vert S_{n} ( t ) - S_{1} ( t ) \bigr\Vert &= \bigl\Vert S_{n} ( t ) - S_{n-1} ( t ) + S_{n} ( t ) - \cdots - S_{1} ( t ) \bigr\Vert \\ &\leq \bigl\Vert S_{n} ( t ) - S_{n-1} ( t ) \bigr\Vert + \bigl\Vert S_{n-1} ( t ) - S_{n-2} ( t ) \bigr\Vert +\cdots + \bigl\Vert S_{2} ( t ) - S_{1} ( t ) \bigr\Vert \\ &= \sum_{i=2}^{n} r_{1}^{i} = \frac{r_{1}^{2} - r_{1}^{n}}{1- r_{1}} \end{aligned}$$

and

50 $$\begin{aligned} \bigl\Vert I_{n} ( t ) - I_{1} ( t ) \bigr\Vert ={}& \bigl\Vert I_{n} ( t ) - I_{n-1} ( t ) + I_{n} ( t ) - \cdots - I_{1} ( t ) \bigr\Vert \\ \leq{}& \bigl\Vert I_{n} ( t ) - I_{n-1} ( t ) \bigr\Vert + \bigl\Vert I_{n-1} ( t ) - I_{n-2} ( t ) \bigr\Vert +\cdots + \bigl\Vert I_{2} ( t ) - I_{1} ( t ) \bigr\Vert \\ ={}& \sum_{i=2}^{n} r_{2}^{i} = \frac{r_{2}^{2} - r_{2}^{n}}{1- r_{1}} . \end{aligned}$$

Since $r_{i} <1$, $S_{n} ( t ), I_{n} ( t )$ are a Cauchy series in $C [ 0,T ]$, and this series is uniformly convergent, it has a unique limit (Adams). So as $n\rightarrow \infty $, in Eq. (18), $S_{n} ( t )$ and $I_{n} ( t )$ have unique fixed points and therefore the system in (7) has a solution and this solution in unique. □

## Numerical results and discussion

In this section, we present the result, for which we use COVID-19 data. The approach developed in [31] is used to approximate the ABC integral. We first use the fundamental theorem on the system (6) and obtain

51 $$\begin{aligned} S ( t ) - S_{0} ={}& \frac{1-\alpha }{X ( \alpha )} M_{1} \bigl( \alpha,t,S ( t ),I ( t ) \bigr) \\ &{} + \frac{\alpha }{X ( \alpha ) \Gamma ( \alpha )} \int _{0}^{t} M_{1} \bigl( \alpha,\tau,S ( \tau ),I ( \tau ) \bigr) ( t - \tau )^{\alpha - 1} \,d\tau \end{aligned}$$

and

52 $$\begin{aligned} I ( t ) - I_{0} ={}& \frac{1-\alpha }{X ( \alpha )} M_{2} \bigl( \alpha,t,S ( t ),I ( t ) \bigr) \\ &{}+ \frac{\alpha }{X ( \alpha ) \Gamma ( \alpha )} \int _{0}^{t} M_{2} \bigl( \alpha,\tau,S ( \tau ),I ( \tau ) \bigr) ( t - \tau )^{\alpha - 1} \,d\tau . \end{aligned}$$

At time $t= t_{m+1}, m=0,1,\ldots $ , we obtain following iterative scheme:

53 $$\begin{aligned} S ( t_{m+1} ) - S_{0} = {}&\frac{1-\alpha }{X ( \alpha )} M_{1} \bigl( \alpha,t,S ( t_{m} ),I ( t_{m} ) \bigr) \\ &{}+ \frac{\alpha }{X ( \alpha ) \Gamma ( \alpha )} \int _{0}^{t} M_{1} \bigl( \alpha,\tau,S ( \tau ),I ( \tau ) \bigr) ( t_{m+1} -\tau )^{\alpha - 1} \,d\tau \\ ={}& \frac{1-\alpha }{X ( \alpha )} M_{1} \bigl( \alpha,t,S ( t_{m} ),I ( t_{m} ) \bigr) \\ &{}+ \frac{\alpha }{X ( \alpha ) \Gamma ( \alpha )} \sum_{j=0}^{m} \int _{t_{j}}^{t_{j+1}} M_{1} \bigl( \alpha,\tau,S ( \tau ),I ( \tau ) \bigr) ( t_{m+1} -\tau )^{\alpha - 1} \,d\tau, \end{aligned}$$

54 $$\begin{aligned} I ( t_{m+1} ) - I_{0} ={}& \frac{1-\alpha }{X ( \alpha )} M_{2} \bigl( \alpha, t_{m},S ( t_{m} ),I ( t_{m} ) \bigr) \\ &{}+ \frac{\alpha }{X ( \alpha ) \Gamma ( \alpha )} \int _{0}^{t} M_{2} \bigl( \alpha,\tau,S ( \tau ),I ( \tau ) \bigr) ( t_{m+1} -\tau )^{\alpha - 1} \,d\tau \\ ={}& \frac{1-\alpha }{X ( \alpha )} M_{2} \bigl( \alpha, t_{m},S ( t_{m} ),I ( t_{m} ) \bigr) \\ &{}+ \frac{\alpha }{X ( \alpha ) \Gamma ( \alpha )} \sum _{j=0}^{m} \int _{t_{j}}^{t_{j+1}} M_{2} \bigl( \alpha,\tau,S ( \tau ),I ( \tau ) \bigr) ( t_{m+1} -\tau )^{\alpha - 1} \,d\tau. \end{aligned}$$

Now, the function $M_{i} ( \alpha,\tau,S ( \tau ),I ( \tau ) )$ is approximated by Lagrange interpolation on $[ t_{n}, t_{n+1} ]$ as

55 $$\begin{aligned} M_{i} \bigl( \alpha,\tau,S ( \tau ),I ( \tau ) \bigr) \cong{}& \frac{ ( \tau - t_{n-1} )}{h} M_{i} \bigl( \alpha, t_{n},S ( t_{n} ),I ( t_{n} ) \bigr) \\ &{}- \frac{ ( \tau - t_{n} )}{h} M_{i} \bigl( \alpha, t_{n-1},S ( t_{n-1} ),I ( t_{n-1} ) \bigr),\quad i=1,2 . \end{aligned}$$

Substituting (55) into (53)–(54) and by integration, we have

56 $$\begin{aligned} S ( t_{m+1} ) - S_{0} ={}& \frac{1-\alpha }{X ( \alpha )} M_{1} \bigl( \alpha,t,S ( t_{m} ),I ( t_{m} ) \bigr) \\ &{}+ \frac{\alpha }{X ( \alpha ) \Gamma ( \alpha )} \Biggl( \sum_{j=0}^{m} \frac{M_{1} ( \alpha, t_{j},S ( t_{j} ),I ( t_{j} ) )}{h} \int _{t_{j}}^{t_{j+1}} ( \tau - t_{j-1} ) ( t_{m+1} -\tau )^{\alpha - 1} \,d\tau \\ &{}- \sum _{j=0}^{m} \frac{M_{1} ( \alpha, t_{j-1},S ( t_{j-1} ),I ( t_{j-1} ) )}{h} \int _{t_{j}}^{t_{j+1}} ( \tau - t_{j} ) ( t_{m+1} -\tau )^{\alpha - 1} \,d\tau \Biggr) \\ ={}& \frac{1-\alpha }{X ( \alpha )} M_{1} \bigl( \alpha,t,S ( t_{m} ),I ( t_{m} ) \bigr) + \frac{\alpha }{X ( \alpha ) \Gamma ( \alpha )} \Biggl( \sum_{j=0}^{m} \frac{h^{\alpha } M_{1} ( \alpha, t_{j},S ( t_{j} ),I ( t_{j} ) )}{\Gamma ( \alpha +2 )} \\ &{}\times\bigl( ( m - j+2 )^{\alpha } ( m+ \alpha - j+2 ) - ( m - j )^{\alpha } ( m+2 \alpha - j+2 ) \bigr) \\ &{}- \frac{h^{\alpha } M_{1} ( \alpha, t_{j-1},S ( t_{j-1} ),I ( t_{j-1} ) )}{\Gamma ( \alpha +2 )} \Biggr) \\ &{}\times \bigl( ( m - j+1 )^{\alpha +1} - ( m - j )^{\alpha } ( m+ \alpha - j+1 ) \bigr), \end{aligned}$$

57 $$\begin{aligned} S ( t_{m+1} ) - S_{0} = {}&\frac{1-\alpha }{X ( \alpha )} M_{1} \bigl( \alpha,t,S ( t_{m} ),I ( t_{m} ) \bigr) \\ &{}+ \frac{\alpha }{X ( \alpha ) \Gamma ( \alpha )} \Biggl( \sum_{j=0}^{m} \frac{M_{1} ( \alpha, t_{j},S ( t_{j} ),I ( t_{j} ) )}{h} \int _{t_{j}}^{t_{j+1}} ( \tau - t_{j-1} ) ( t_{m+1} -\tau )^{\alpha - 1} \,d\tau \\ &{} - \sum _{j=0}^{m} \frac{M_{1} ( \alpha, t_{j-1},S ( t_{j-1} ),I ( t_{j-1} ) )}{h} \int _{t_{j}}^{t_{j+1}} ( \tau - t_{j} ) ( t_{m+1} -\tau )^{\alpha - 1} \,d\tau \Biggr) \\ ={}& \frac{1-\alpha }{X ( \alpha )} M_{1} \bigl( \alpha,t,S ( t_{m} ),I ( t_{m} ) \bigr) + \frac{\alpha }{X ( \alpha ) \Gamma ( \alpha )} \Biggl( \sum_{j=0}^{m} \frac{h^{\alpha } M_{1} ( \alpha, t_{j},S ( t_{j} ),I ( t_{j} ) )}{\Gamma ( \alpha +2 )} \\ &{}\times \bigl( ( m - j+2 )^{\alpha } ( m+ \alpha - j+2 ) - ( m - j )^{\alpha } ( m+2 \alpha - j+2 ) \bigr) \\ &{}- \frac{h^{\alpha } M_{1} ( \alpha, t_{j-1},S ( t_{j-1} ),I ( t_{j-1} ) )}{\Gamma ( \alpha +2 )} \Biggr) \\ &{}\times\bigl( ( m - j+1 )^{\alpha +1} - ( m - j )^{\alpha } ( m+ \alpha - j+1 ) \bigr). \end{aligned}$$

### Simulation

*Case I: The world*

**Figure 2:**
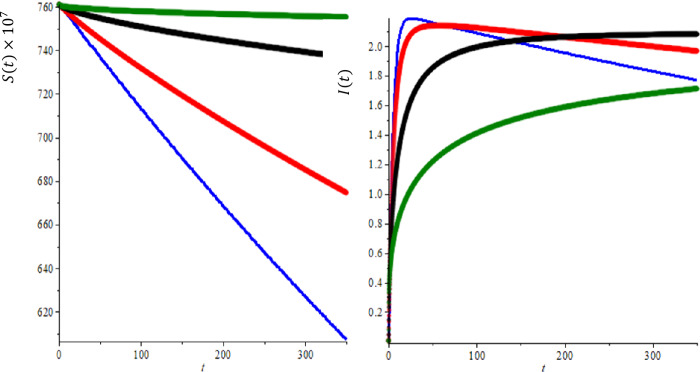
Comparison of the results of integer order derivatives (blue line, $\alpha =1$), fractional order of derivatives ($\alpha =0.9$ (red line), $\alpha =0.7$ (black line) and $\alpha =0.5$ (green line) for $b=100$ and $R_{0} =2.618$

**Figure 3:**
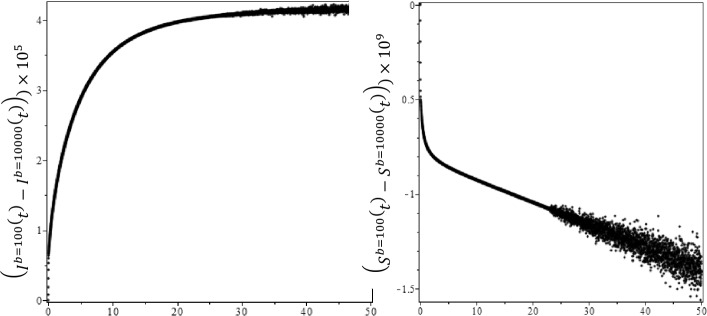
The differences of active infected cases and suspected individuals for $b=100$ and $b=10{,}000$

We need to use real data for COVID-19 to determine the parameters, the current birth rate for the world in 2020 is 18.077 births per 1000 people, and the death rate is 7.612 per 1000 people [16]. The world’s population on February was $N = 7{,}610{,}105{,}452$, so $A= \frac{n\times N}{365} =391{,}347, \mu = \frac{7.612}{365\times 1000} =2.08547\times 10^{-5}$, according the WHO report [32] the COVID-19 mortality rate $\gamma =0.034$. The other parameters can be found by a curve fitting technique with real reported data for COVID-19. Figure 1(a) shows the development of susceptible individuals decrease with the time as expected for integer-order derivatives, $b=100$ and $R_{0} =2.618$. Figure 1(b) shows the variations of infected individuals with the time, it increases with time and reaches the plateau region as seen in each country, where we used same parameters as Fig. 1(a). We compare the prediction fractional-order derivatives in Fig. 2. It is well known that an increase in the number of active cases and reaching the plateau region are different for each country and the number of suspected population decreases and reaches the steady case. This is also different for each country. On the other hand, from Fig. 2, it is clear that the active or suspected population not only is a function of time but also a function of the fractional order. This fact proves that the fractional-order derivative can be used the adjust the modeling to the different countries. The other point for fractional-order derivative can also be used as a free parameter to fix the modeling with the data. The effect of the hospital beds for controlling of the diseases are given in Fig. 3(a)–(b). Figure 3(a) shows the difference of the results between $b=100$ and $b=10{,}000$. In Fig. 3(a) we show the infected individuals’ difference between $b=100$ and $b=10{,}000$ could reach 400,000 daily cases, if we assume that only 20% of infected individuals need medical treatment at hospital, then, if we multiply this one by COVID-19 mortality rate 0.034, we obtain 2720 people life can be saved if we provide sufficient hospital beds. Figure 3(b) shows the difference of the suspected individual results of $b=100$ and $b=10{,}000$, this is expected because active infected cases for $b=100$ always are bigger than active infected cases for $b=10{,}000$. We also note that our numerical results converge to the disease equilibrium point.

*Case 2: Saudi Arabia*

**Figure 4:**
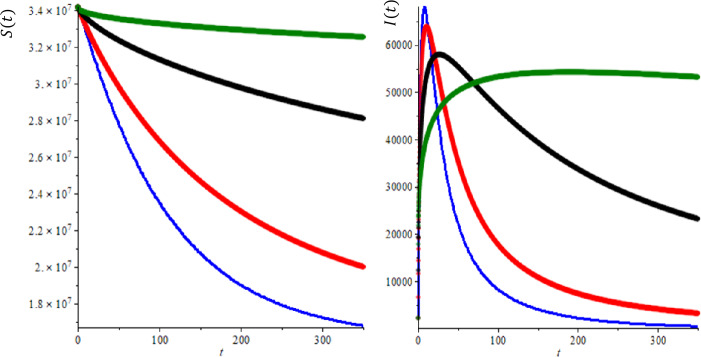
Comparison of between the results of integer order derivatives (blue line, $\alpha =1$), fractional order of derivatives ($\alpha =0.9$ (red line), $\alpha =0.7$ (black line) and $\alpha =0.5$ (green line) for $b=100$, $k=5000$ and $R_{0} =2.618$

**Figure 5:**
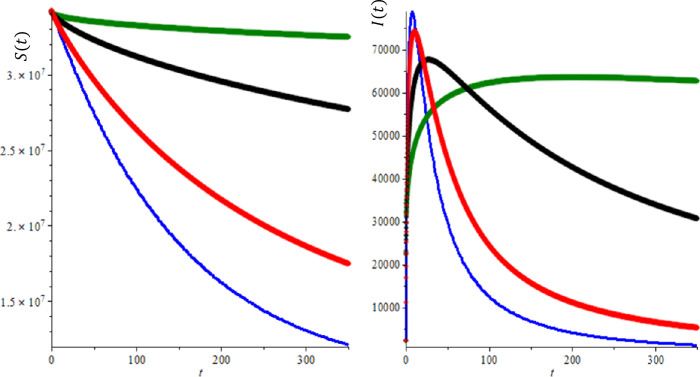
Comparison of between the results of integer order derivatives (blue line, $\alpha =1$), fractional order of derivatives ($\alpha =0.9$ (red line), $\alpha =0.7$ (black line) and $\alpha =0.5$ (green line) for $b=100$, $k=3000$ and $R_{0} =3.565$

In the second case, we provide a numerical simulation using real data for the transmission model of COVID-19 in Saudi Arabia. According to the WHO report, the total population of Saudi Arabia $N =34{,}218{,}169$ so $A= \frac{n\times N}{365} = \frac{0.01335\times 3{,}418{,}169}{365} =1252, \mu = \frac{0.0133564}{365} =3.6593\times 10^{-5}$, we assumed COVID-19 mortality rate $\gamma =0.034$. On April 8, $I(0)=2260$ and $S(0)=34{,}218{,}169$. In Fig. 4(b), we explored the prediction of active cases for different values of fractional order, $\alpha =1$ (blue line), 0.9 (red line), 0.7 (black line) and 0.5 (green line), when we compare modeling prediction with the real data (WHO report active cases in Saudi Arabia [32]), we see that model prediction overestimate sometimes lower estimate, but again it seems that $\alpha =0.9$ produces the best result. At this point, we note that even our suggested modeling seems simple, but we believe that prediction of this model can be used as a basis for more complicated model predictions. Furthermore, we have one more adjustable parameters in fractional-order derivatives, this gives us enormous flexibility. Further, in Fig. 5, we concentrate on the effect of half saturation constant *k* over the suspected and infected individual for several values of fractional order of the derivations. We can see as before the effect of the fractional order provides us with enough flexibility and suspected individuals decrease with time and eventually reach the equilibrium point as we expected again the time to reach the equilibrium point to depend on the fractional order of the derivative.

## Conclusion

In this study, consideration is given to a complex mathematical fractional SIR model for spreading of the COVID-19 virus, we first show the feasibility region where the solution exists. The equilibrium points (disease-free and endemic equilibrium) of the system are found. The local and global asymptotic stability of the system are discussed. Using the fixed-point theorem, existence and uniqueness of solution is proven. A numerical solution of the system is performed for several values of the parameters involved in the modeling. We showed the importance of maintaining a sufficient number of hospital beds. We also discussed and compared the prediction of our modeling with real word data. Finally, we concluded that using the fractional derivative provides us with one more parameter, which can be used to simulate the real word data.

## Data Availability

The authors confirm that the data supporting the findings of this study are available within the article [and/or] its supplementary materials.
